# Live Streams on Twitch Help Viewers Cope With Difficult Periods in Life

**DOI:** 10.3389/fpsyg.2020.586975

**Published:** 2020-11-20

**Authors:** Jan de Wit, Alicia van der Kraan, Joep Theeuwes

**Affiliations:** Department of Communication and Cognition, Tilburg Center for Cognition and Communication, Tilburg School of Humanities and Digital Sciences, Tilburg University, Tilburg, Netherlands

**Keywords:** live streaming, difficult periods in life, coping, game culture, mental well-being, human-media interaction

## Abstract

Live streaming platforms such as Twitch that facilitate participatory online communities have become an integral part of game culture. Users of these platforms are predominantly teenagers and young adults, who increasingly spend time socializing online rather than offline. This shift to online behavior can be a double-edged sword when coping with difficult periods in life such as relationship issues, the death of a loved one, or job loss. On the one hand, platforms such as Twitch offer pleasure, distraction, and relatedness with others to help with coping, and the increased sense of anonymity and control could stimulate self-disclosure. However, the prevalence of trolling and memes may also discourage people from opening up, and relationships that are built online—especially those with microcelebrity streamers—could be perceived as more meaningful than they actually are. To create a deeper understanding of Twitch as a new media platform embedded in game culture, and how users perceive its potential as a coping mechanism, we have conducted a first exploration by means of a survey. The questions focused on general Twitch behavior, the difficult period in life, and the role of Twitch during this period. It was distributed online among people who considered themselves active Twitch users, and who had gone through a difficult period. Eighty-four participants completed the entire survey. The majority of participants indicated that Twitch helped them cope, and that it became a larger part of their lives during the difficult period compared to regular viewing. Recurring themes were the entertainment, distraction, and sense of community Twitch offers. Viewing behavior during difficult periods appears to remain largely the same in terms of the streamers that are watched, although time spent viewing increases, and there is a change toward more time spent actively watching rather than having the stream on in the background. With this work, we aim to create a deeper understanding of Twitch as a platform, and its importance for gamers that are going through difficult periods in life.

## 1. Introduction

Game culture has grown beyond actively playing games, as an increasing amount of game play is being broadcast online. Live streaming services such as Twitch provide ways for a large audience to spectate others as they play, while simultaneously being able to interact with the broadcaster and with other viewers, all from the comfort of their own homes. The rise in popularity of live streaming started in the early 2010's with the broadcasting of esports tournaments, and has since grown into an industry in which individual *streamers* (broadcasters) have made it their full-time job to provide entertainment for an audience of up to tens of thousands of viewers on a daily basis. Platforms such as Twitch are “complex and rich ecosystems” (Deng et al., 2015, p. 1), that are increasingly being studied through various lenses such as media and game studies, psychology, and communication. The multidisciplinary nature of research into live streaming is perhaps best captured by Taylor's description of the phenomenon as “an interesting collision of the televisual, computer games, the internet, and computer-mediated communication” (Taylor, 2018, p. 2).

Twitch has become a popular pastime, with the average user spending 95 min per day watching live streams on the platform. The majority of people active on Twitch are male (81.5%), and 55% of users are between 18 and 34 years old, with the average age being 21 years old^1^. This constitutes an age group in which the transition from adolescence into adulthood takes place (Arnett, 2014, as cited in Mahmoud et al. 2012). This transition generally includes major life changes such as moving out of the parental home and becoming increasingly independent, starting college or working life, (romantic) relationships developing and evolving, and trying to find one's identity and purpose in life. Not being able to successfully complete these transitional steps, as well as the general academic, social, and financial stress stemming from life as a student, can lead to anxiety and depression (Mahmoud et al., 2012). We can therefore assume that a substantial part of the people active on Twitch are going through this transitional phase, and could be experiencing mental health issues as a result. In addition, there are a number of invasive life events that could happen at any age, such as divorce, the death of a loved one, coming out, or job loss, which could also have a detrimental effect on people's mental well-being.

There are several ways, both constructive (adaptive) and destructive (maladaptive), to cope with these difficult periods caused by life events or by transitioning into adulthood. A maladaptive coping strategy such as avoidance may make things worse and in fact lead to exacerbation of the mental health issues (Mahmoud et al., 2012). With especially the youth spending an increasing amount of time online (Lenhart et al., 2010), on platforms such as Twitch, we feel that it is important to investigate if these media are being used as coping mechanisms during difficult periods in life and, if this is the case, whether this is an adaptive or maladaptive form of coping. Although an increasing amount of research is being done into Twitch and similar live streaming platforms, to our knowledge the potential role of Twitch as a coping mechanism during difficult times has not yet been explored.

By means of a survey study, we set out to investigate whether Twitch is perceived as a useful coping mechanism by its users, and how this is supported by the different elements of this ecosystem. For example, we attempt to find out to what extent the televisual and computer game aspects of live streaming provide a sense of distraction, and how the computer-mediated social aspects could lead to tight-knit, safe communities in which meaningful conversations take place. We want to emphasize that we do not intend to promote Twitch as a viable alternative to professional help. Instead, we consider it a potential complement to treatment, a way to lower the threshold to seek professional help, and a platform that can be used—and is already being used—to reduce the stigma surrounding mental health.

## 2. Background

### 2.1. Live Streaming and Twitch

Live streaming platforms are participatory online communities in which users can provide content by broadcasting a video feed, which can then be consumed by other users acting as viewers. At the same time, a chat facility running parallel to the video broadcast enables viewers to interact with the broadcasters, also known as streamers, and with each other in real-time (Hamilton et al., 2014; Taylor, 2018). Twitch is currently the largest live streaming platform, with over 2 million viewers and over 90,000 broadcasts active on average at any time^2^. It originally started as the “Gaming” category of the general live streaming platform Justin.tv, which was founded in 2007. In 2011, this section containing broadcasts of people playing games had grown into its own dedicated platform, Twitch. Amazon bought Twitch in 2014 for $970 million. Live streaming is different from other user generated content (UGC) platforms, such as YouTube, mainly due to the active participation of viewers and streamers (Gandolfi, 2016), and the similarities it shares with live sports platforms (Deng et al., 2015). Next to individual streamers that often broadcast according to fixed schedules and are generally receiving consistent viewership, there are certain esports and charity events that temporarily attract large numbers of viewers. While these events tend to take on a more traditional top-down broadcasting style, the individual streamers often allow the viewers to become part of the show, for example by responding to their input from the chat, or by inviting them to join games that are being played on stream (Gandolfi, 2016).

**Figure 1:**
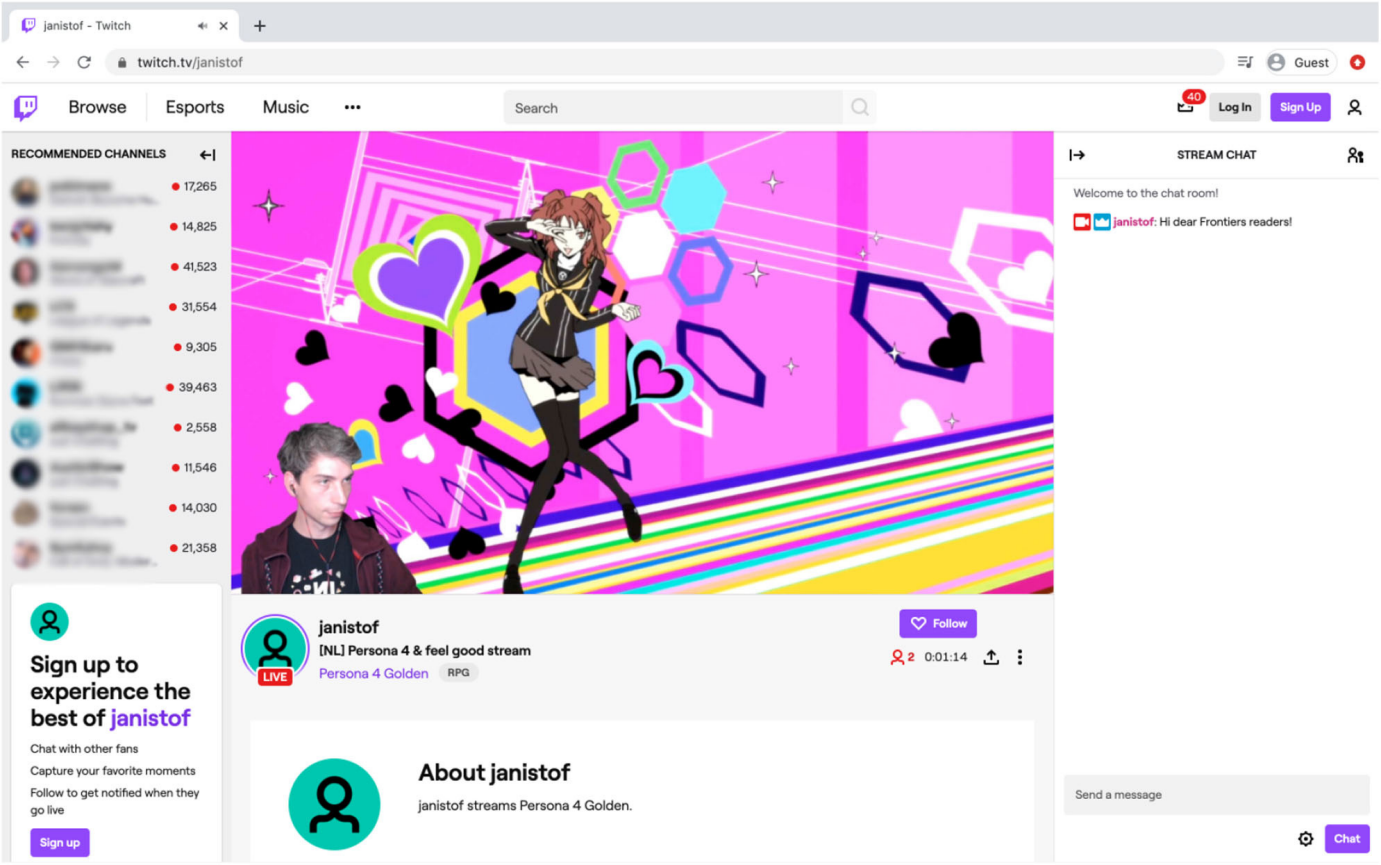
Twitch from the viewer's perspective (not logged in). On the left is a list of popular channel suggestions (blurred for privacy), in the middle the broadcasted game and streamer, on the right the chat. Image taken with permission (the streamer is the first author of this paper).

Twitch contains several design features that shift the focus from the game being played onto the streamers and viewers present on the platform (Anderson, 2017), some of which are depicted in the viewer's perspective of a broadcast shown in Figure 1. For example, people create their own identity though their user names and channel pages, and the streamer is usually visible and audible on the stream alongside the game being played (Sjöblom et al., 2019). Multiple sources have found that most viewers choose which channels they watch based on the streamer, instead of the games that are being played (Hamilton et al., 2014; Deng et al., 2015; Gandolfi, 2016; Anderson, 2017). This move toward focusing more on the person rather than the game was taken even further in 2016 when Twitch introduced the *In Real Life (IRL)* category of streams, and relaxed the requirements on streams having to center around game play. This category was later succeeded by a number of more specific variations, including *Just chatting, Sports and fitness*, among others. The chat is another key feature that allows the live streaming audience to become visible and actively participate, turning them into a community with shared experiences instead of passive, individual viewers (Hamilton et al., 2014). The main reasons for people to engage with live streams are tension release, social interaction and community, entertainment, and information seeking (Gandolfi, 2016; Sjöblom and Hamari, 2017; Hilvert-Bruce et al., 2018; Taylor, 2018).

Gandolfi (2016) distinguishes, based on theories of immersion, between three different orientations of a streamer toward their viewers, which determines the amount of interaction that takes place between them: (1) as professional, which focuses on the streamer's expertise at challenging and competitive games, with little to no interaction with viewers; (2) as hedonist, where the viewers experience a game's aesthetics through the streamer, usually without competitive pressure, sometimes in the context of emergent game play such as speed running^3^ or open-ended (sandbox) experiences and role play. In these streams there tends to be little interaction with viewers, and it is mostly about the game being played; (3) as companion, which is more about the streamer as a person rather than an extension of the game avatar. These streams have a large degree of interaction between the streamer and the viewers, and the topics of conversation go beyond the game that is being played. It is possible for the same streamer to switch orientations, even mid-stream, for example when a professional competitive player takes time as a companion to interact with the audience at the start of the stream before loading the game, or when they decide to pick up a casual, non-competitive game and take on a hedonic role.

Streamers make money from playing advertisements, sponsorship deals, contracts with live streaming platforms and game publishers, and donations from viewers (Johnson and Woodcock, 2017). Viewers can subscribe to a streamer at $4.99 for one month, part of which goes to the streamer and part to Twitch. The viewer receives several benefits in return, such as an icon next to their username and additional emotes (emoji) to be used in chat. They can also make one-time donations. Both types of donations can usually be accompanied by a message to the streamer, which is often automatically displayed on stream and acknowledged by the streamer (Sjöblom et al., 2019). A number of streamers have managed to make enough money to make live streaming their main occupation. Wohn et al. (2018) have found that, in addition to making donations, viewers also provide emotional and instrumental support to the streamers. An important predictor of viewer loyalty, in terms of repeated viewing behavior and how much support is given to streamers, is whether or not viewers develop a parasocial relationship with the streamer (Hamilton et al., 2014; Hu et al., 2017; Lim et al., 2020). A parasocial relationship is a one-sided intimate connection with a media performer such as a streamer, based on repeated encounters (Dibble et al., 2016). This relationship is one-sided as the streamer does not experience this connection with, nor gives the same amount of support to the viewer. The parasocial relationship, in turn, is stimulated by wishful identification, which means that viewers picture the streamer as a role model and want to imitate them (Hoffner and Buchanan, 2005), as well as by emotional engagement with the streamer and with other viewers watching the same broadcast (Hamilton et al., 2014; Lim et al., 2020).

The distribution of viewers among channels is highly skewed, with the top 1% of channels attracting 70% of all viewers (Deng et al., 2015). This means that there is a small group of popular streamers that attract tens of thousands of concurrent viewers, while most streamers have only several hundreds of viewers or less, or even no viewers at all. Hamilton et al. (2014) considers streams with over 1,000 concurrent viewers “massive,” and notes that in these streams, it can be difficult for the streamer and the viewers to keep up with the fast pace at which messages appear in the chat, which the researchers compare to the dynamics of a stadium crowd (cf. the similarity between live streaming and live sports viewing dynamics suggested by Deng et al. 2015). As a result, more meaningful interactions tend to take place in streams with smaller audiences (Nakandala et al., 2017; Hilvert-Bruce et al., 2018). However, this does not mean massive streams are only watched for the games being played, because the streamer is still valued as an entertainer, regardless of the lack of direct communication (Hamilton et al., 2014). It is also possible for the streamer to reduce the pace of the chat, by allowing only subscribers (viewers that have donated the $4.99 fee for a month) to post messages. This enables meaningful conversation to take place in larger streams, although at the cost of accessibility (Hamilton et al., 2014).

Twitch is used predominantly by young people, with an average age of 21 years old and 55% of the users between the ages of 18 and 34 years old^4^. A vast majority (81.5%) of users is male, which is also reflected in the large number of channels featuring male streamers, compared to female streamers (Nakandala et al., 2017). Nakandala et al. (2017) have found differences in the types of conversation that take place depending on the gender of the streamer. The chat of popular male streamers (with large numbers of viewers) contains more game-related words, while the chat of popular female streamers tends to have more objectifying words (i.e., focusing on the streamer's appearance, or considering them an object rather than a person). These differences are not present in smaller streams, although more social signaling words were found in smaller channels featuring a female streamer. This can be seen as an indication that these streams are considered more as a social gathering than a sports event (Nakandala et al., 2017).

### 2.2. Difficult Periods in Life and Coping

In our study, we asked participants to reflect on a difficult period in their lives. Although most people can intuitively describe what is meant by a difficult period and name examples such as divorce, abuse, loss of a loved one, or transitioning into college life, formal definitions of what constitutes a difficult period may vary. Herron et al. (2016) call these “sensitive life experiences” and describe them as life events and transitions that put individuals in a vulnerable state. Massimi et al. (2012) talk about “life disruptions,” and call the vulnerable state they leave people in “destabilizing.” In addition, these disruptive events are said to be unpredictable and uncontrollable. The effects on people are often invisible to outsiders, stigmatized, and last for a prolonged period of time (Massimi et al., 2012). Folkman and Lazarus (1984) describe the resulting feeling of “stress” as a combination of personal characteristics and an event that happens in the person's environment. This means that there are individual differences in the impact of the same potentially stressful event, based on personal characteristics (i.e., resilience, past experiences). In their research on the role of playing games during difficult times, Iacovides and Mekler (2019) adopt a definition from Pals (2006), which describes a difficult time as anything that is perceived by the person as stressful, confusing, troubling, or discouraging. This captures several of the elements mentioned previously, such as the vulnerable and destabilized state, and the unpredictable and uncontrollable aspects of the event or transition (resulting in confusion and discouragement).

Coping is defined by Folkman and Lazarus (1984) as “constantly changing cognitive and behavioral efforts to manage specific external and/or internal demands that are appraised as taxing or exceeding the resources of the person.” This definition focuses on coping as a process, rather than a personality trait. Different coping processes can be categorized as either problem-focused or emotion-focused, where problem-focused processes are commonly used if the underlying stressors can be changed or controlled, and emotion-focused processes when the stressor cannot be addressed. Problem-focused strategies can be focused on the environment (e.g., removing barriers, finding resources) or on the self (e.g., learning new skills). The majority of emotion-focused strategies is aimed at reducing the emotional distress caused by the stressor, including avoidance, minimization, and distancing. Combinations of both types of strategies can prove to be beneficial, for example by first caring for one's emotional well-being before addressing the underlying cause with a problem-focused approach. However, they can also negatively affect each other, e.g., when one gets stuck in an unproductive cycle of a problem-focused coping strategy (such as seeking additional information) that only leads to increased feelings of emotional distress (Folkman and Lazarus, 1984). A further distinction can be made between constructive (adaptive) and destructive (maladaptive) coping strategies (Mahmoud et al., 2012). However, which strategy is considered adaptive or maladaptive is highly context-dependent. For example, although avoidance (escapism) is generally considered a maladaptive coping strategy, it can be helpful to first distance oneself from the stressor or to avoid a direct confrontation, before addressing it with a problem-focused coping strategy (Folkman and Lazarus, 1984).

Life transitions, such as moving out of the parental home and starting college life, can lead to difficult periods and are common in early adulthood (18–24 years old), an age group that also makes up a substantial part of the Twitch users. This time of adolescence and early adulthood is also when we try to find out who we are and develop an identity, and when we attempt to engage in intimate, long-term relationships (Erikson and Erikson, 1998). Additionally, people going through puberty and young adulthood (approximately 10–25 years old) are said to use more maladaptive coping strategies than other age groups (Mahmoud et al., 2012). Furthermore, research has suggested that there are differences in coping strategies between men and women, where women are generally more likely to turn to emotion-focused strategies and seek social support, while men tend to opt for problem-focused strategies but are also more likely to resort to alcohol or drugs as an (emotional) avoidance strategy (Ptacek et al., 1994). In addition, particularly younger men have relatively low mental health literacy and are less likely to seek professional help compared to women (Rice et al., 2018).

### 2.3. Game Culture and Coping With Difficult Periods in Life

The present study is inspired by the work of Iacovides and Mekler (2019), in which they investigated by means of a survey how actively playing games could be beneficial during difficult times. They found that games were indeed commonly used as a coping mechanism. Games are able to offer players ways to distract themselves, to confront their feelings, to connect with others, and to achieve personal growth. These coping strategies appear to be both emotion-focused (e.g., distraction, connecting with others) as well as problem-focused (e.g., confronting feelings, personal growth). This is congruent with the observed social and emotional benefits of playing games (e.g., Olson, 2010; Granic et al., 2014; Kowert et al., 2014). However, participants in the study also highlighted several negative effects of spending too much time playing games, such as a decrease in productivity, physical activity, or real-life socializing. Games were referred to by some participants as a maladaptive coping strategy, mostly in the sense that they could be used to avoid confronting the underlying stressors for a prolonged period of time. There is an ongoing debate about the use of games as an avoidance (escapism) strategy. Games offer a way for people to gather their bearings, to recover, and to regulate their moods before confronting the issues at hand (Kosa and Uysal, 2020). However, because games are designed to be highly engaging there is a pitfall of escaping for too long, beyond the point where one should be confronting their difficulties (Griffiths, 2015; Kosa and Uysal, 2020).

When comparing game play to watching others play, we envision similarities as well as differences between the experiences, benefits, as well as challenges offered. Although a sense of agency is lost because the player becomes an observer, the presence of the streamer and the possibility of interacting with other viewers add new sources of entertainment and community. This is what Gandolfi (2016) refers to as the “reverse remediation” of live streaming, turning game play into video while extending the original game into a public and social setting. Most notably, the chat adds the option of also discussing serious and personal topics that are not related to the game being played. The anonymity of Twitch, combined with the lighthearted nature of game play, could lead to levels of self-disclosure similar to or exceeding those commonly found in online social communication (Valkenburg and Peter, 2009).

Although the specific role of live streaming platforms such as Twitch in coping with difficult periods has not previously been researched, several related research avenues do indicate that there are people active on the platform that are going through a difficult time, and that Twitch has had a beneficial effect on them. For instance, in Wohn et al. (2018) viewers are quoted saying they received valuable help from streamers as well as from other viewers. This was a reason for them to provide (monetary) support to the streamer. Hilvert-Bruce et al. (2018) found that a lack of external support in real life served as a motivator for engaging more with live streams. They highlight the potential of using online communities as a way to fight loneliness, and as a safe alternative for people who struggle to engage in real-life social interactions. Participants in the study by Iacovides and Mekler (2019) also indicated that they were involved with other aspects of game culture, such as live streaming, and that the act of becoming a streamer provided social support.

We imagine that, compared to the general population, a relatively large number of streamers will have experienced, or is currently experiencing difficulties themselves. Most streamers are adolescents or young adults that are going through the same life transitions as the majority of their viewers. In addition, life as a streamer can be stressful and competitive in its own right (Johnson and Woodcock, 2017), so it may well be that building a career out of live streaming at a relatively young age is already a stressful life transition. Finally, live streaming can be a viable profession for people with certain mental or physical conditions, such as social anxiety, that prevent them from participating in jobs that require them to leave the house (Johnson, 2018). The parasocial relationships that form between these streamers and their viewers could put them in the position of a role model, allowing them to improve mental health awareness and literacy by opening up about their own struggles. In fact, several streamers have taken up the role as mental health advocates^5^, and Twitch has responded to this by creating a *Mental health* category of streams. The fact that Twitch is aware of streamers and viewers going through difficult times is also apparent from their dedicated page with mental health support and information^6^, which was developed in collaboration with Take This, a mental health organization that is active in the gaming community^7^.

Considering the reported motivations for viewers to engage with Twitch—tension release or distraction, entertainment (emotion regulation), and social interactions—we see a potential role of Twitch as a coping mechanism. We expect that this role is further supported by the design of the platform, which places an emphasis on the people present on Twitch, and facilitates the development of communities. In addition, the parasocial relationship with the streamer and the emotional engagement with other viewers may provide social support, and stimulate self-disclosure by viewers that are going through difficult times. Knowing that smaller communities, and specifically those centered around female streamers, tend to have more social and meaningful communication, we aim to investigate whether viewers show a preference toward these types of broadcasts during their difficult times, and whether their perceived relationships with the streamer and other viewers change. Finally, we explore whether female viewers adopt different coping strategies on Twitch compared to male viewers.

## 3. Methodology

We conducted an exploratory study into the use of Twitch, and how viewers' behavior on the platform might change during difficult periods in life, by means of a survey. Due to the sensitive nature of the questions, the participants were well-informed that they could stop filling in the survey at any time if they felt uncomfortable doing so. The data were collected in November–December 2019.

### 3.1. Measures

A survey was created and implemented in Qualtrics. It included a total of 30 questions from existing literature on Twitch viewing behavior (Gandolfi, 2016; Gros et al., 2017), and on the effects of playing games during difficult periods in life (Iacovides and Mekler, 2019), as well as questions that were designed specifically for the current study. The survey consisted of four parts: (1) demographic information; (2) general video game playing and Twitch viewing behavior; (3) description of the experienced difficult period in life; (4) Twitch viewing behavior during the difficult period in life. The questions were a combination of open-ended questions, multiple choice questions, and Likert scale ratings. The full list of questions can be found in the Supplementary Materials.

The demographics questions included age, gender, level of education, and country of birth. These questions were included to verify, by comparing with existing data on the Twitch user base, whether the participants in the current study represent the general viewer population on Twitch, or whether a particular subset can be identified that is more likely to use Twitch as a coping mechanism. Furthermore, the inclusion of these questions allowed us to explore whether there were any differences regarding viewing behavior on Twitch, or in terms of coping strategies, based on factors such as gender identity.

Participants were then asked to describe their general gaming behavior, as well as their activities on Twitch. These questions were included to find out whether they see themselves as gamers, and how important watching live streams is to them. We based these questions on literature that looked into live streaming viewership, including elements that motivate people to engage on platforms such as Twitch (Gandolfi, 2016; Gros et al., 2017). We included additional questions to investigate whether participants were more likely to watch streams with many or few viewers, and female or male streamers. Finally, participants were asked to describe how they use Twitch, for example whether they only observe streams or also interact with the streamer or other viewers. There is no mention of a difficult period in life at this point, in order to establish a baseline of general viewing behavior. It is possible that some participants never experienced Twitch outside of their difficult period, although we would still expect the severity of their difficulties to fluctuate which could lead to changes in behavior as the severity increases.

At the start of the section regarding the difficult period in life, participants were reminded once more that they were free to stop their participation at any time. A definition of what was considered a difficult period was then presented, which was identical to the description used by Iacovides and Mekler (2019) in their research on playing games during difficult periods in life, but where references to *playing games* were replaced with *watching Twitch*. The description, which was originally based on Pals (2006), was as follows:

All of us have times of—perhaps ongoing—personal difficulty. Please think of stressful, confusing, troubled, or discouraging time in your life, during which you watched Twitch. Please describe this difficult time in your life. What did you experience as stressful, confusing, troubling, or discouraging?

Participants were then asked to categorize this difficult period (e.g., depression, physical health), and were given the option to provide a more elaborate description. Although details regarding the difficult periods in life that our participants experienced were not necessary in order to investigate whether Twitch can serve as a coping mechanism, these questions were still included in the survey for three reasons. Firstly, it allowed us to verify that the experience described by the participant aligned with the provided definition of a difficult time. If this turned out not to be the case, we would be able to exclude participants or revise our definition. Secondly, these descriptions help to create a deeper understanding of what participants have been through, allow us to empathize with them, and they add context to participants' descriptions of the role of Twitch during this difficult period. Finally, this section acted as a segue to the last section of the survey, concerning the role of Twitch during this difficult period.

The final part of the survey started with a question about the amount of time spent watching Twitch during the difficult period in life, and whether participants perceived watching Twitch to be helpful during this period. Depending on the answers to these questions, participants were directed to a different set of follow-up questions. Participants who did not watch Twitch at all, or who did not find it helpful, were asked whether they hypothetically could see Twitch as being helpful and, if so, how. The other participants, who did indicate that they watched Twitch during the difficult period and stated that it was at least somewhat helpful (scores 3–5 on a 5-point Likert scale) received follow-up questions regarding their Twitch behavior during this time, for example related to stream size and their interactions on the platform. This allowed us to compare the behavior during the difficult period with the baseline provided by the earlier set of questions. We also asked them to recall and describe a specific event in which Twitch was helpful to them, to provide context for the (mostly quantitative) description of the behavior on Twitch.

The survey was iteratively reviewed and revised, with support from colleagues in our department. It was then pilot tested with three participants, who were familiar with Twitch but did not necessarily experience a difficult period, so they were asked to imagine that they did. The survey was found to be clear during the pilot test, the only minor change that was made was to add a genre of games to the list of options (*battle royale*).

### 3.2. Participants

Participants were recruited through voluntary response sampling, by advertising the survey on various subreddits (r/Samplesize, r/Twitch, r/Mentalhealth, r/Greekgodx, r/Reckful, and r/Forsen), and Facebook groups related to Twitch. The survey was also retweeted by JJ Balderok, a Dutch game journalist with approximately 9,000 followers, and it was discussed in chat on several Twitch channels, which resulted in a streamer on the Belgian *Kayzr* channel (approximately 5,600 followers) discussing the research during their broadcast. Two selection criteria were communicated when advertising the survey, which were that participants needed to have been active as viewers on Twitch, and they had to have gone through, or were still going through, a difficult period in life. This may have resulted in a sample that is not representative of the Twitch viewership in general, however we deliberately chose these criteria because our main focus was on exploring if and how Twitch could be used as a coping mechanism, not on creating a quantitative overview of the number of people on Twitch that had experienced a difficult time.

A total of 128 participants started the survey. Out of these, six were deleted because they only provided answers to the questions about demographics, and did not complete the second part on their interactions with Twitch. Another nine were deleted because they showed satisficing behavior (i.e., straightlining), or because the replies to the open-ended questions included nonsense, were clearly trolling, or referred to memes. This is an inherent part of doing research online, especially in popular culture, and we were even warned that this might happen by some of the serious participants. Out of the 113 remaining participants, 84 completed the entire survey. For clarity and consistency, we included only these 84 participants when discussing the results.

All participants gave informed consent, and the research was approved by the Research Ethics and Data Management Committee (REDC) of the Tilburg School of Humanities and Digital Sciences, at Tilburg University. Participants were issued a random identifier at the start of the survey, which was used to pseudonymize the data. The data collection and storage procedures are compliant with the General Data Protection Regulation (GDPR). There was no compensation for participation in the survey.

### 3.3. Procedure

The survey could be opened and completed remotely, on a computer or on a mobile device. The first page introduced the study, and reminded participants of the inclusion criteria (they had to be users of Twitch, and have experienced a difficult period). After giving informed consent, the participants went through the four sections of the survey in the order as described in section 3.1. Care was taken to ensure them that they could stop their participation at any time. It took participants 3–99 min to complete the survey, with an average of 16 min (*SD* = 16 min). One participant was excluded when measuring the completion time because they left the browser tab open overnight. After completing the survey, participants were thanked for their time, and were asked to leave their e-mail address if they would like to be contacted for potential follow-up questions. Because the questionnaire responses already provided rich and comprehensive data, we decided not to contact any participants. Following the example of Iacovides and Mekler (2019), the subreddit and Facebook pages where the survey was posted were monitored in case participants voiced any concerns, and the researchers also had regular debriefing meetings while analyzing the data.

### 3.4. Analysis

Due to the exploratory nature of the current study, no statistical testing was performed on the quantitative responses to the multiple choice and Likert scale questions. Instead, we use descriptive statistics and graphs to identify and illustrate patterns that occur in these data.

The responses to the open-ended questions were coded by two researchers, using a bottom-up approach. After coding the data independently, the resulting codes for each question were compared and merged into a final set, and the responses to the open-ended question were coded with this set. There were cases in which multiple codes applied, for example in the descriptions of the difficult times that participants went through, where there could be multiple elements to the difficult period (e.g., physical health problems and depression). In this case, we took an inclusive approach and assigned both codes, without making any assumptions about how different codes might relate to each other.

## 4. Results

### 4.1. Demographic Information

Out of the 84 participants that completed the survey, nine identified as female, 73 as male, 1 as other, and 1 did not want to disclose this information. The average age was 23 years (*SD*= 6 years). Participants were from 30 different countries (1 unknown), with most participants born in the Netherlands (*N* = 23), the United States of America (*N* = 10), the United Kingdom (*N* = 6), Canada (*N* = 5), and Germany (*N* = 5).

### 4.2. General Video Game and Twitch Behavior

**Figure 2:**
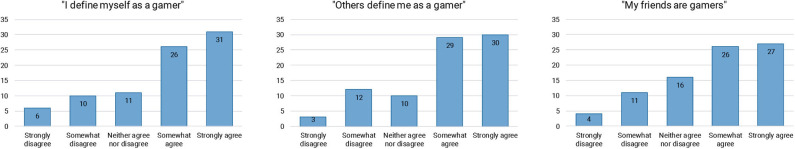
Answers to questions investigating whether participants consider themselves part of game culture.

To get an overview of whether Twitch viewers consider themselves as being part of game culture, we asked them to indicate on a five-point Likert scale whether they define themselves as a gamer, whether others define them as gamers, and whether their friends are gamers. As shown in Figure 2, most participants consider themselves part of game culture.

**Figure 3:**
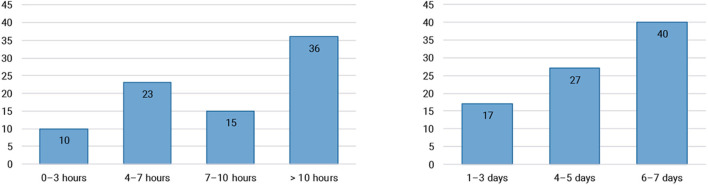
Weekly viewing patterns on Twitch, in hours per week **(left)** and days per week **(right)**.

We were also interested to see how big the role of Twitch is in people's lives. This was measured by asking how many hours per week, and on how many days of the week, people tend to watch Twitch. The results (see Figure 3) indicate that Twitch is a common pastime, with the majority of respondents watching more than ten hours a week, spread across 6 or 7 days.

#### 4.2.1. Motivations and Preferences for Watching

**Figure 4:**
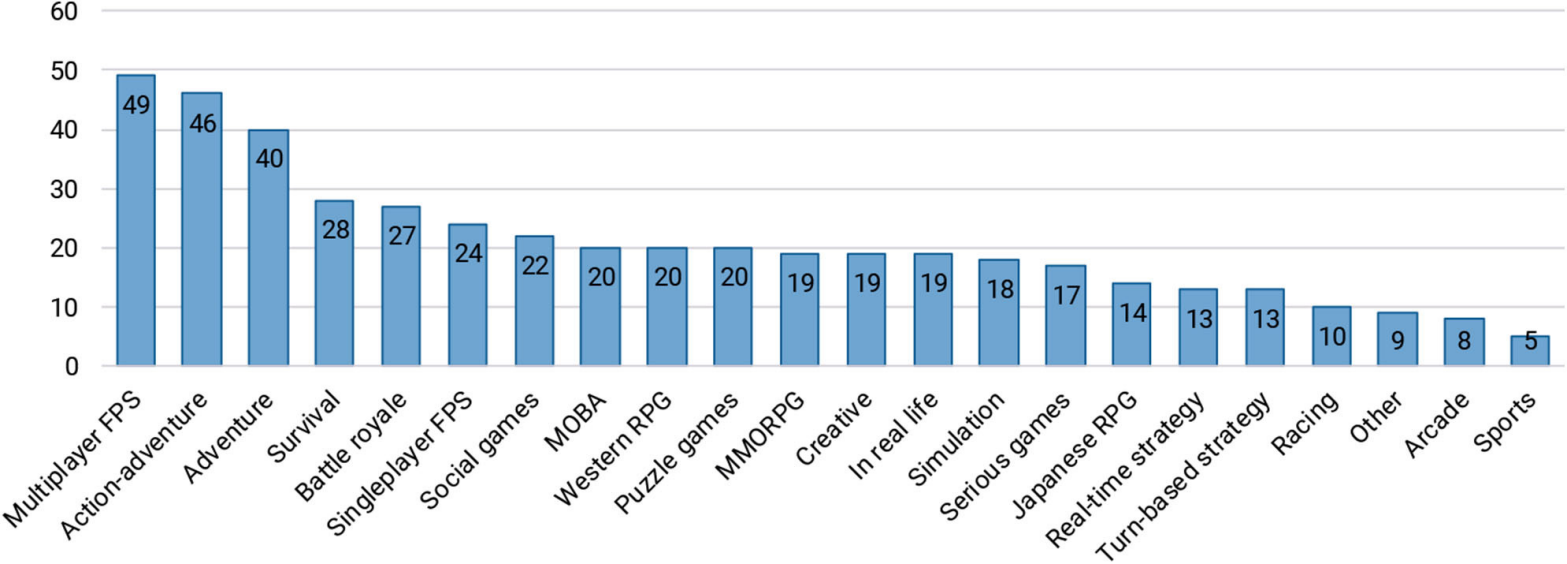
Participants' preferred game genres to watch (they were able to select multiple answers).

There is a broad range of different genres being broadcast on Twitch, so we asked participants which types of games they preferred watching (Figure 4). Two categories of streaming channels that are not directly related to gaming, *Creative* and *In Real Life*, were also included. Participants that assigned *Other* referred to collectible card games, low quality games, and live events such as LAN parties. Furthermore, three participants indicated that they choose which channels to watch based on the streamer, and not the game being played. We also asked whether the games that participants tend to watch on Twitch are the same as the games that they play themselves, with which 33 people (39%) either disagreed or strongly disagreed.

**Figure 5:**
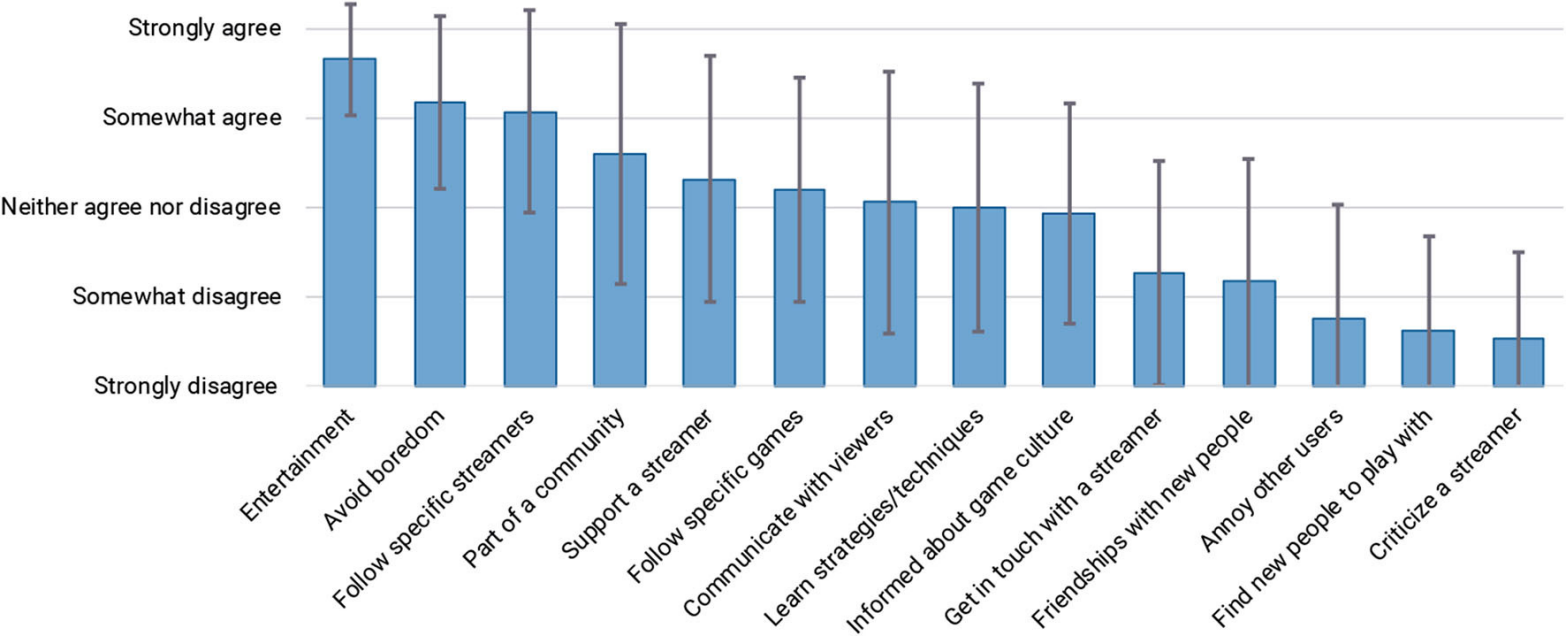
Main motivations for watching Twitch.

It appears that to several viewers, the streamer and their community are more important in keeping them engaged than the game that is being played. This is further supported by participants' answers to the question about their main reasons for using Twitch, shown in Figure 5. The main motivations for watching live streams appear to be entertainment, and the ability to follow streamers and become part of their community.

To test our assumption that people would prefer to turn to smaller, more intimate communities that allow for more personal interaction, we asked participants about their preferences regarding stream size. Based on literature (Hamilton et al., 2014), we distinguished between smaller streams (1,000 or less concurrent viewers), and larger streams (above 1,000 concurrent viewers). This baseline could then be compared with their preferences during the difficult period in life. Seventeen participants (20%) indicated that they watched smaller streams, while 28 participants (33%) preferred larger streams, and the remaining 39 (46%) watched a combination of small and large streams.

Furthermore, we asked how often the participants watched male or female streamers. The possible answers were never, sometimes, about half the time, most of the time, always, or I don't know. Male streamers were watched at least about half the time by all respondents (1 indicated they did not know), where 77 participants (92%) indicated to spend most of the time or all their time watching male streamers. In contrast, 66 participants (79%) indicated to never or only sometimes watch a female streamer (1 indicated they did not know). However, these results should be interpreted with caution, because there is also a substantially larger number of male streamers on the platform. In other words, these results do not necessarily indicate a preference toward male streamers, they could simply be a result of female streamers being underrepresented on Twitch. If we look at the 9 female participants and 73 male participants in the study separately, we do see that 5 of the female participants (63%) watch female streamers at least half the time, while this only applies to 12 male participants (16%).

#### 4.2.2. Interactions on Twitch

In the final part of this section, we inquired about the use of various features on the platform, such as the ability to chat with other viewers, and to interact with the streamer. All participants indicated that there was some degree of interaction between the streamer and the viewers, with 62 participants (74%) reporting a lot of, or even constant interaction. However, the strength of their personal connection to the streamer was more diverse, with 19 participants (23%) reporting a very weak to weak connection, 38 participants (45%) considering their personal connection average, and 27 participants (32%) indicating a strong to very strong connection to the streamer. Regarding the interactions with other viewers, we asked participants how often they were reading the chat, and how often they wrote messages of their own. While the majority (64 participants, or 76%) indicated to read the chat either most of the time or always, only 35 participants (42%) actively participated themselves most of the time or always. Another 36 participants (43%) indicated that they never or only sometimes contribute to the chat. The reported personal connection to other viewers is similarly spread out as the connection to the streamer, where 26 participants (31%) reported a very weak to weak connection, 30 participants (36%) having an average connection, and 28 participants (33%) forming a strong or very strong bond with other viewers.

Finally, an open-ended question was included to find out what participants would usually do on Twitch, for example whether they were only watching, commenting on the stream, or talking about personal things. This also provided insight into the contexts in which people watch Twitch, as 34 participants indicated that they would at times have the stream on in the background, while they were in fact doing other things (eating, chores, playing games themselves). When participating in chat, 38 participants indicated that stream-related topics were discussed, while 17 participants also interacted about other things, such as details regarding their personal lives. Multiple participants mentioned spamming memes, such as typical Twitch emotes or catchphrases, as their contribution to the chat. One participant highlighted how it can be challenging to get a word in, if the chat is very active.

### 4.3. Difficult Period

**Figure 6:**
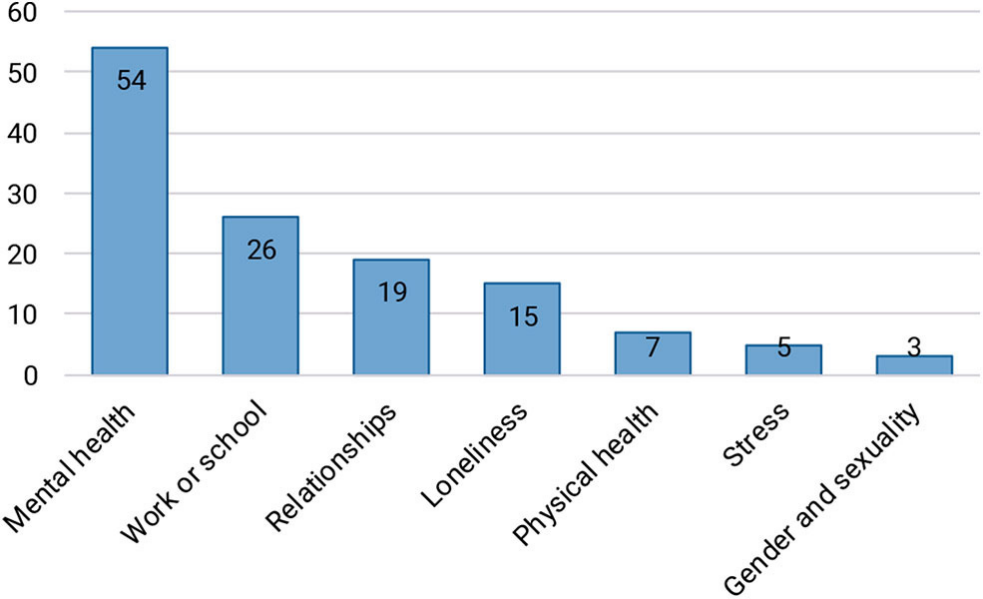
Types of difficult periods encountered by participants. It is possible for one participant to have reported multiple categories. Relationships can refer to romantic partners, family, or friends.

The survey continued by asking participants about the difficult period in life they had experienced. A definition of a difficult period was provided, after which participants were asked to recall and then categorize the difficult times they experienced. Several examples of these categories were provided, such as depression, relationship issues, and physical health issues. Subsequently, there was space to provide a more elaborate description of the difficult period, however this was not required. The answers to both open-ended questions were coded in order to identify recurring themes, and the number of participants that mentioned each theme is shown in Figure 6. Note that it was common for one participant to have experienced a combination of these codes. A large number of participants experienced a form of depression or (social) anxiety, often combined with—or triggered by—other difficulties.

How several factors can be interconnected, and how this difficult period can persist for a prolonged period of time, is illustrated by the following participant:

I sometimes get depressed because of my medical problems that I've had to deal with since I was born. Which makes me have low self esteem. Which then makes it hard for me to socialize. Family problems, moving to different states & countries constantly and having to make new friends all the time etc. [...] (P82, 18, male)

The majority of the participants in the study were young adults, an age group that is characterized by several potentially impactful life changes, such as moving out of the parental home, romantic relationships becoming more serious, and transitioning to higher education or to working life. This could lead to stress or mental health problems:

due to a new point in my schooling and moving out on my own but still being financially dependent on family, I've felt trapped within my new space. (P109, 20, female)

Thirty-nine participants (46%) indicated that they were currently still in this difficult period in life, while 29 (35%) no longer were. An additional 14 participants (17%) were unsure, and 2 (2%) did not wish to disclose this information. In the remainder of section 4, we do not distinguish between participants that were currently still in the difficult period in life, and those that were no longer in this period. However, it is important to keep in mind the variation in the nature of the difficult period, as well as the stage participants were currently in.

### 4.4. Twitch During the Difficult Period

Six participants did not watch Twitch during the difficult period in their lives, and were therefore not asked about any changes in their behavior on Twitch during the period. Instead, they were asked whether they thought Twitch could potentially be helpful in difficult times. Another nine participants did watch Twitch, but did not find it to be helpful. One participant illustrates this with an example:

Finishing a master's degree comes with pressure. Twitch is the place to relax. By visiting Twitch I waste more time which should be invested in my thesis. Resulting into more stress, more twitch and so on... and so on... (P1, 24, male)

The same participant did indicate that, although Twitch was not helpful to them in this particular case, it could be helpful in dealing with difficult periods in general, because you can find people that are similar to yourself on the platform. We assume that, in the particular case of having to finish the master's degree, the most efficient way to get out of the difficult period was to commit to getting the work done, and Twitch would be an unconstructive distraction. Another participant expressed concerns about using Twitch to cope with difficult periods, as it may even exacerbate the problems that people are experiencing:

Twitch is a dangerous source of “social” activity. Its ease of access can lead to cutting off with the real world. As a result of this risk, Twitch is not a good source of help during a difficult period in life. (P17, 26, male)

Most people, however, were positive about watching Twitch during their difficult period, as 29 out of the 69 participants that used Twitch during this time found it to be very helpful (42%), 20 participants (29%) found it to be helpful, and 20 participants (29%) found it to be somewhat helpful to them. We asked participants to explain what Twitch meant for them during their difficult period, and—optionally—to recall a specific situation when Twitch was particularly helpful to them. One participant, who was struggling with their own sexuality, remembered a specific event involving a monetary donation to the streamer by another viewer. In most streams, these donations can be joined by a text message, which is then often displayed automatically on the stream, without any moderation or intervention by the streamer. This particular donation contained a homophobic message, which was then instantly shut down by the streamer. In other words, the streamer publicly disapproved of this behavior, which left a powerful positive impression on this participant.

**Figure 7:**
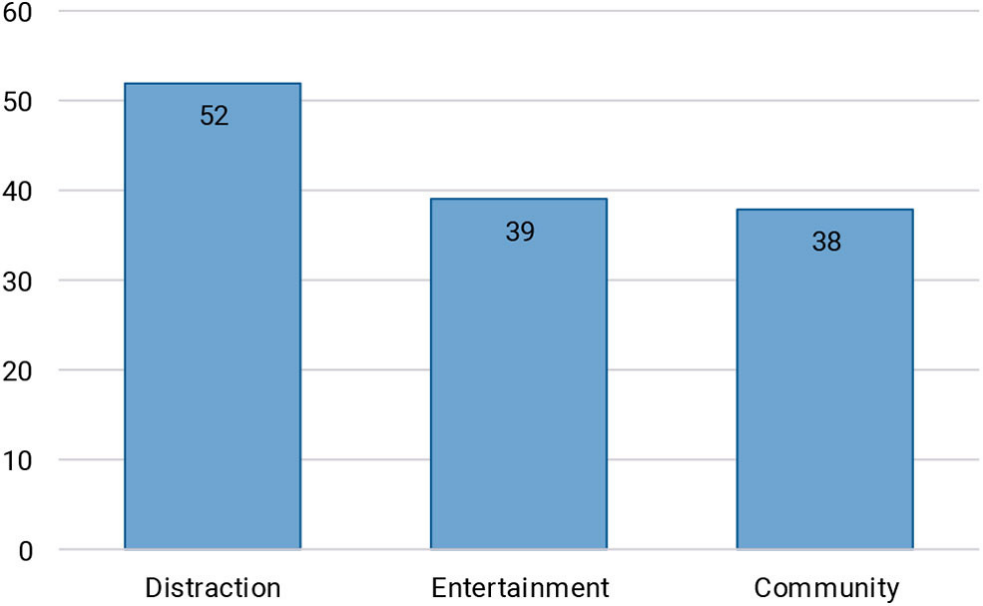
The number of participants that mentioned distraction, entertainment, and being part of a community as the positive effects of watching Twitch during their difficult period. It is possible for one participant to mention more than one theme.

The answers to these two questions were analyzed and three main themes emerged, explaining the positive contributions of Twitch: distraction, entertainment, and being part of a community. Figure 7 shows the number of times these themes were mentioned by participants. It was possible for one participant to talk about multiple themes. There were several gender-based differences regarding entertainment (mentioned by 61% of male participants and 38% of female participants) and community (mentioned by 54% of male participants and 63% of female participants), although with only eight female participants more research is needed to confirm these findings. Distraction was mentioned by approximately the same number of male and female participants (76 and 75%, respectively).

#### 4.4.1. Distraction

For many participants, watching Twitch was a way to temporarily keep their mind off the difficulties they were facing. Watching a live broadcast was considered to be a stronger distraction than watching a prerecorded show:

During many evenings I felt very anxious and could not calm down, I was afraid of nightmares in the night. Watching streams and just knowing the other person is really there talking at the moment helped much better to calm me down than just watching videos on YouTube (P107, 28, female)

The ability to communicate by means of spamming “copy/pastas,” memes, or emoticons, is also seen as a way to distract oneself from negative thoughts:

I made a copypasta on Avast's stream and chat liked it and copied it. It was a very happy moment where I felt proud of something, even if it was a stupid meme. It meant something to me. (P46, 19, male)

This spamming can be a method for viewers to share in an emotional experience with the streamer, for example when certain key events happen in the game (Hamilton et al., 2014). Even if it may not be a solution to their problems, being present in a community on Twitch, hearing someone speak and sharing the experiences made participants feel less lonely. For some, Twitch was something to look forward to in a time when they had little else to look forward to. Participants also mentioned that Twitch offered stability, as it was “[...] something constant in [their] life in a period where [they] felt everything is changing” (P107, 28, female).

#### 4.4.2. Entertainment

A number of participants reported not only finding distraction in live streams, but also thoroughly enjoying, and being cheered up by watching them. Streamers as entertainers bring a positive attitude that inspires the viewers and provides them with a more optimistic outlook on life:

I was stressed, didn't know what to do with my life, I just wanted to be alone all the time. Then I found tyler1 and greekgodx playing games together (overwatch, minecraft etc.) and their interaction always made me chuckle and brightened my day. I am forever thankful for that. (P54, 24, male)

Greekgodx in particular was lauded by several participants for his positive attitude, and his ability to make people laugh. In addition to the streamers' positive attitude, viewers get a sense of joy out of the community as a whole:

The streamers that have a strong community and their own “inside jokes” and that just genuinely have a fun and entertaining streams. To name a few; forsen, xqcOW. (P90, 22, male)

#### 4.4.3. Community

There are different ways in which viewers can get involved in the streamer's community. There are streamers that have sessions where they play games with viewers, or invite guests to participate in a talk show-like setting. Furthermore, streamers can also view and respond to the chat, and they tend to acknowledge donations or people subscribing to their channel on stream. Several participants indicated that Twitch can serve as a safer, less stressful environment for social interactions:

It definitely gave me a feeling of positivity and having a community. It feels like you are hanging out with the streamer but not having the social pressure that you usually have when you hang out with friends etc. (P90, 22, male)

In most responses to these open-ended questions, there was a clear distinction between the real and the virtual. At the same time, some participants indicated that by engaging in social interactions online, in relative anonymity, they experienced personal growth that transferred into real life:

Talking to people helped a lot with my social anxiety. As I mentioned, even the simplest interactions could stress me out. As I started interacting with the streamers I liked to watch and chatting to the other viewers, I found myself growing more comfortable with talking to strangers and being myself online as well as in real life. It's a wonderful distraction too, and something to look forward too which can motivate me throughout the day. Belonging to a community is definitely a big part of it as well. (P103, 19, female)

One participant specifically mentions the use of the *offline chat*, a design feature that we had previously not considered in this research. When streamers are not broadcasting, their page and the chat remain active. This allows visitors to interact with each other, and be an active part of the streamer's community, even if the streamer is not around. It also enables popular streamers to provide not only entertainment and distraction through their live broadcasting, and through fast-paced interactions in the chat (e.g., spamming), but also to build a tight-knit community with more in-depth, serious conversation using the offline chat and other platforms such as Discord or social media. Several participants found a community in which they felt comfortable discussing personal matters, and were able to receive emotional support in return. At times, it is even the streamer who opens up about their own difficulties first:

I'm not going to name the streamer because they were talking about their problems during this stream, but the way they expressed completely matched how I was feeling. I was surprised that there was someone else feeling what I was feeling, but then people in chat started to step forward and claimed they also felt the same way. Some got passed the “feeling” they had and shared what worked for them and even offered to be a line of emotional support if needed later. (P48, 22, male)

Another participant mentioned a format that was introduced by popular streamer Pokimane, called *Dr. Poki*. In these segments, Pokimane invites viewers to “call in” (through voice chat), to discuss their real life issues with her on stream. At the same time, memes and jokes are being posted in the chat in rapid succession, which adds a light-hearted touch to the otherwise relatively serious talk happening on stream. In addition, people felt accepted by the communities on Twitch:

[...] it's really nice to know that somewhere, even though it's somewhere that doesn't exist physically (a chatroom) I can still feel accepted even if I don't feel accepted into society at large (P113, 22, did not wish to disclose).

#### 4.4.4. Changes in Viewing Behavior

In order to investigate whether Twitch became a bigger part of participants' lives, we asked them whether they thought they spent more, less, or an equal amount of time watching Twitch during the difficult period in their lives, compared to their viewing behavior outside of this difficult period. Fifty participants (72%) indicated that they spent at least a little more time watching Twitch (1 did not recall). Furthermore, based on the assumption that it is easier to find emotional support in smaller communities, we repeated the previous question about participants' preferred stream sizes, now during the difficult period. However, there was no significant shift in preferred stream sizes compared to general viewing behavior (22% small streams, 38% large streams, 40% both sizes). There was also no change in the amount of time participants spent watching female streamers compared to male streamers. In general, it appears that participants kept watching the same streams during the difficult period that they were already watching outside of this period.

Participants also did not report a stronger personal connection to other viewers (*M*_*dif*_ = 0.13, *SD* = 0.77 on a five-point Likert scale), nor to the streamer (*M*_*dif*_ = 0.13, *SD* = 0.97) as they were going through their difficult period. For the eight female participants in our study, this increase in the strength of the personal connection with other viewers (*M*_*dif*_ = 0.38, *SD* = 0.74), and with the streamer (*M*_*dif*_ = 0.38, *SD* = 0.52) was larger than for the male participants. The perceived amount of interaction between the streamer and the viewers did not increase (*M*_*dif*_ = 0.07, *SD* = 0.71). However, although participants did not send more messages to chat than before (*M*_*dif*_ = −0.03, *SD* = 0.82), they did indicate reading chat more during the difficult period (*M*_*dif*_ = 0.20, *SD* = 0.70). This may be caused by a shift toward more active viewing, rather than having a live stream on in the background.

## 5. Discussion

We set out to explore whether live streaming platforms such as Twitch could offer support for viewers going through difficult times, such as relationship issues, the death of a loved one, or job loss. A survey containing multiple choice questions, rating scales, and open-ended questions was created and distributed through a number of channels related to gaming and Twitch live streaming. Eighty-four participants contributed by completing the entire survey and provided us with rich and extensive descriptions of their experiences. It was impossible for us to tell all of their stories, but we hope to have provided a clear impression.

Before discussing the nature of the difficult period, and how Twitch had potentially provided support during this period, we asked several questions about the participants' general experiences with live streaming. We found that most participants consider themselves to be part of game culture, and that they spend a substantial part of their time watching Twitch. The main motivation for watching Twitch is to be entertained, and to follow specific streamers and to be part of their community. We found that the streamer and their community are more important than the game that is being played (cf. Deng et al., 2015; Gandolfi, 2016; Anderson, 2017). This was further supported by the fact that many participants indicated that they watch different game genres than the ones they actually play themselves. Furthermore, most of the streamers that the participants watched tend to interact frequently with their viewers, for example by addressing their comments in the chat. These findings regarding Twitch viewing behavior are generally consistent with the survey by Gandolfi (2016). We did find that participants in our study spent more time watching live streams, compared to the findings in Gandolfi (2016). This could be due to differences in recruitment strategies—we recruited mostly in Twitch-related networks, while Gandolfi (2016) recruited in game-related networks—or due to the rise in popularity of Twitch over the past years.

Most participants either prefer to watch larger streams (of at least 1,000 concurrent viewers), or a combination of larger and smaller streams. The majority of streamers that were mentioned in the answers to open-ended comments as having played an important role for participants during their difficult times—e.g., Lirik, Forsen, xQcOW, Pokimane, and Greekgodx—are popular streamers with over 1,000 viewers. Male streamers are more commonly watched than female streamers, although this could be due to the larger number of male streamers present on Twitch. It did seem to be the case that female participants in our study watched more female streamers than the male participants did, however with only nine female participants we cannot draw strong conclusions. The skewed gender distribution in our sample is a result of the voluntary response sampling approach combined with the over-representation of viewers that identify as male on Twitch (Nakandala et al., 2017). In future work we are interested in specifically studying differences in viewing behavior, effects, and preferences based on gender. There were large individual differences in how strong the perceived connection with the streamer and with other viewers was. An interesting finding was that a number of people used Twitch as something to have on in the background while they engaged in other activities such as playing games themselves, or completing various chores.

After learning more about the participants' Twitch viewing behavior, we made an inventory of the types of difficult periods they had encountered, and found that the majority of cases were related to problems with mental health. The difficulties were often complex and long-term, which we assume is why most participants (46%) were still going through this difficult period when they completed the survey. A vast majority of participants (82%) found Twitch to be at least somewhat helpful during their difficult times. Perhaps as a result of this, they also reported spending more time on Twitch while experiencing these difficulties. Other than increasing their time investment, participants' viewing behavior did not change much. Although we expected that it would be possible to get more emotional support in smaller streams, it appears that people prefer to stay with the streamers that they were already watching before they were going through a difficult time. We also discovered several ways for larger communities to tap into some of the advantages of smaller communities, such as using offline chat and other communication platforms outside of Twitch, or featuring viewers on the stream (e.g., by playing games with them). At the same time, smaller communities still appear to provide more in-depth conversation and possibilities to interact with the streamer, and some participants indicated that they shift their viewing behavior based on their needs:

Either watching LIDL^8^ games with Forsen for entertainment or spending times in smaller communities and talking with other viewers and the streamer (P96, 23, male)

One additional, subtle change in viewing behavior we observed was that participants reported reading the chat more while they were experiencing their difficult time, compared to their general Twitch behavior. We believe this might be an indication that they started spending more time actively watching, rather than having the stream on in the background.

Out of all of the participants' reports on how Twitch was helpful to them during their difficult times, we distilled three main themes: distraction, entertainment, and community. Much like when actively playing games (Iacovides and Mekler, 2019), watching live streams can keep your mind off the difficulties. This can be done by immersing oneself in the streamer's gameplay, or by engaging with the streamer and the chat, even if simply by spamming memes. Participants also reported events of being entertained, and seeing their mood improve by watching live streams. This stems mostly from the commentary and behavior of the streamer and other viewers. Finally, by becoming part of a streamer's community the viewers were able to reduce their feelings of loneliness, get a sense of belonging, engage socially with others, and in some cases even talk openly about their struggles. A main advantage of online communities such as those on Twitch, compared to socializing offline, is that viewers get to choose to what extent they want to hold on to their anonymity, and how much they self-disclose. There is less pressure, and fewer expectations:

The idea of being apart of a community of people anonymously and not having to talk about myself and everyone being okay with how little/much they shared about themselves. I guess I looked forward to log on twitch and see everyone again. (P48, 22, male)

These three themes—distraction, entertainment, and community—align well with coping strategies that people use in general when dealing with difficult times (Folkman and Lazarus, 1984). The majority of coping strategies used on Twitch appear to be emotion-focused, mostly adaptive, with some problem-focused strategies such as practicing social skills as well. As one participant did note, there are situations such as having to finish schoolwork in which Twitch cannot serve as an adaptive coping mechanism, and instead poses a risk of distracting people from the task at hand. Literature has also shown that there are individual differences regarding the preferred coping strategy, for example based on gender (Ptacek et al., 1994). Although we see some hints toward differences in the use of Twitch as a coping mechanism based on gender (e.g., female participants watching more female streamers, and reporting more social benefits and a stronger connection to other viewers and streamers during the difficult time, compared to male participants), future research with a more balanced sample is needed to verify these phenomena. It appears that with the ability to distract, entertain, and provide social connections, Twitch streaming has something to offer for most people, and it is possible to seek out particular streamers based on one's preferences. For example, those looking for a larger degree of interaction with the streamer could seek channels where the streamer acts as a companion, while those wanting to be distracted by expert game play could opt for streamers that take on the role of a professional (cf. Gandolfi, 2016).

Although we did not specifically investigate the nature of the relationship with the streamer, it does appear that wishful identification and parasocial relationships occur between viewers and streamers, as reported in Lim et al. (2020). For example, one participant mentioned living vicariously through the streamer, and it has become clear from our studies as well that viewers tend to be loyal to particular streamers and their communities, which was also found by Wohn et al. (2018). Several researchers indicate that learning how to play, or get better at certain games is a motivation for watching a stream (Hamilton et al., 2014; Lim et al., 2020), although this motivation did not score highly in the present study. Lim et al. (2020) indicate that this is a form of observational learning, a process in which one learns by observing a (role) model (in this case, streamers) exhibit a behavior (a game play strategy) and the response they receive in return (e.g., a reward in the form of victory or score points) (Bandura, 2001). We postulate that observational learning might also happen with streamers' social behavior, and their approach to managing their own mental well-being. Several streamers have been through a difficult period themselves, and the job of being a streamer can be stressful (Johnson and Woodcock, 2017; Johnson, 2018). Therefore, we believe that streamers, especially those that self-disclose about their own struggles, can serve as a role model for viewers and facilitate observational learning of skills other than gaming. In the future, we would like to look deeper into the nature of this relationship between a streamer and their viewers. As one of the participants in the study also indicated, there is a risk that the relationships with the streamer and with other viewers on Twitch become a replacement for genuine, real world relationships with others. However, several people indicated that, due to their depression or anxiety, it was simply impossible for them to build and maintain these real world relationships. For these people, Twitch was a way to have at least some form of a social connection, and in some cases it even served as a safe environment for practicing social skills, which helped build the confidence and comfort to start engaging in real world interactions again. In addition, the participants in our study seemed well aware of the difference between the virtual and the real world, and the one-sided nature of the relationship between themselves and the streamer.

To summarize, we discovered that people did find support on Twitch during difficult periods in their lives. This support tends to come in the form of distraction, entertainment, and being part of a community. These elements are inherent to Twitch as a platform, and to the content that is provided by streamers. As a result, we do not see a large shift in viewing behavior as viewers go through a difficult period, other than an increase in viewing time and a tendency toward more active rather than passive viewing. There are subtle design elements of Twitch (e.g., offline chat), and ways in which streamers behave (e.g., interacting with viewers, providing, and upholding community values) that further enhance the supportive role of Twitch. It is important to reiterate the exploratory nature of the current study. The use of a retrospective self-report comes with certain advantages (e.g., anonymity and richness in information of responses), but also has its limitations, including various biases and inaccuracies such as socially desirable responses and a tendency to agree with what is stated or asked (Paulhus and Vazire, 2007). It is also possible that participants' self-reported experiences and time spent watching were subject to negativity bias (Rozin and Royzman, 2001), or that their capacity for accurate reporting was affected by their current mental state, since approximately half of the participants were still going through a difficult period when filling out the survey. At the same time, watching Twitch could lead viewers to reach a flow state (Csikszentmihalyi, 2000), which could make them understate the amount of time spent. The study can—and should—therefore be complemented with other approaches, such as longitudinal studies, to provide a more accurate description of the relationship between viewers' behavior on Twitch and their emotional state. In addition, it would be interesting to study more deeply the interactions that happen on live streaming platforms, e.g., by means of observations. The strength of the current approach lies in the multitude of potential future research avenues inspired by the qualitative accounts of our participants' experiences, and in the fact that it provided us with insight into the participants' personal lives, including thoughts and feelings that would likely not have been disclosed in observations of live streaming sessions.

## 6. Conclusion

Our aim with this study was to create a more elaborate understanding of the behavior of existing Twitch viewers, and how this may change during difficult times. It is clear from our research that streamers, supported by platforms such as Twitch, do serve an important purpose for people that are active on the platform and are going through a difficult time, even without consciously attempting to do so. They do this by providing a distraction through entertainment, maintaining a positive attitude, and building a tight-knit community around themselves. The majority of Twitch viewers is at an age where they can encounter a number of important life events, which can cause stress and difficulty coping. It is also a demographic that is unlikely to seek out professional help when needed. We have to keep in mind that the interactions that happen on Twitch are not a replacement for any kind of professional support. At the same time, we do believe that the streamers and viewers active on Twitch are able to contribute to mental health awareness and literacy, and hope that they are able to remove some of the obstacles that prevent people from seeking out additional help when needed.

## Data Availability Statement

The datasets presented in this article are not readily available because, due to privacy considerations and the sensitive nature of this topic, participants were not asked to give consent for publishing the raw data. Requests to access the datasets should be directed to Jan de Wit, j.m.s.dewit@tilburguniversity.edu.

## Ethics Statement

The studies involving human participants were reviewed and approved by Research Ethics and Data Management Committee (REDC) of the Tilburg School of Humanities and Digital Sciences, at Tilburg University. Written informed consent from the participants' legal guardian/next of kin was not required to participate in this study in accordance with the national legislation and the institutional requirements.

## Author Contributions

JW, AK, and JT designed the study. AK and JT performed data collection and analysis, and each wrote a master's thesis on this research. JW reviewed both theses and compiled them into a first version of the manuscript, which was then reviewed by AK and JT. All authors contributed to the article and approved the submitted version.

## Conflict of Interest

The authors declare that the research was conducted in the absence of any commercial or financial relationships that could be construed as a potential conflict of interest.

## Footnotes

^1^https://muchneeded.com/twitch-statistics/

^2^https://twitchtracker.com/statistics/

^3^Trying to complete a game as fast as possible, sometimes with specific constraints and sometimes with the use of bugs or exploits.

^4^https://muchneeded.com/twitch-statistics/

^5^A number of streamers that openly discuss mental health are listed as Take This Ambassadors: https://www.takethis.org/programs/ambassador-program/

^6^https://help.twitch.tv/s/article/twitch-cares-mental-health-support-information

^7^https://www.takethis.org/

^8^Low budget games, a term coined by streamer Forsen.

## References

[B1] Anderson S. (2017). Watching people is not a game: interactive online corporeality, Twitch.tv and videogame streams. Game Stud. 17, 1–16. 19058092

[B2] Arnett J. J. (2014). Emerging Adulthood: The Winding Road From the Late Teens Through the Twenties. New York, NY: Oxford University Press. 10.1093/acprof:oso/9780199929382.001.0001

[B3] Bandura A. (2001). Social cognitive theory of mass communication. Media Psychol. 3, 265–299. 10.1207/S1532785XMEP0303_03

[B4] Csikszentmihalyi M. (2000). Beyond Boredom and Anxiety. San Francisco, CA: Jossey-Bass 10.1037/10516-164

[B5] Deng J. Cuadrado F. Tyson G. Uhlig S. (2015). Behind the game: exploring the Twitch streaming platform, in 2015 International Workshop on Network and Systems Support for Games (NetGames) (Zagreb), 1–6. 10.1109/NetGames.2015.7382994

[B6] Dibble J. L. Hartmann T. Rosaen S. F. (2016). Parasocial interaction and parasocial relationship: conceptual clarification and a critical assessment of measures. Hum. Commun. Res. 42, 21–44. 10.1111/hcre.12063

[B7] Erikson E. H. Erikson J. M. (1998). The Life Cycle Completed (Extended Version). New York, NY: WW Norton & Company.

[B8] Folkman S. Lazarus R. S. (1984). Stress, Appraisal, and Coping. New York, NY: Springer Publishing Company.

[B9] Gandolfi E. (2016). To watch or to play, it is in the game: the game culture on Twitch.tv among performers, plays and audiences. J. Gam. Virt. Worlds 8, 63–82. 10.1386/jgvw.8.1.63_1

[B10] Granic I. Lobel A. Engels R. C. (2014). The benefits of playing video games. Am. Psychol. 69:66. 10.1037/a0034857 24295515

[B11] Griffiths M. D. (2015). Gaming addiction and internet gaming disorder, in The Video Game Debate: Unravelling the Physical, Social, and Psychological Effects of Video Games, eds Kowert R Quandt T. (New York, NY: Routledge). 10.4324/9781315736495-5

[B12] Gros D. Wanner B. Hackenholt A. Zawadzki P. Knautz K. (2017). World of streaming. motivation and gratification on Twitch, in International Conference on Social Computing and Social Media (Vancouver, BC: Springer), 44–57. 10.1007/978-3-319-58559-8_5

[B13] Hamilton W. A. Garretson O. Kerne A. (2014). Streaming on Twitch: fostering participatory communities of play within live mixed media, in Proceedings of the SIGCHI Conference on Human Factors in Computing Systems (Toronto, ON), 1315–1324. 10.1145/2556288.2557048

[B14] Herron D. Andalibi N. Haimson O. Moncur W. van den Hoven E. (2016). HCI and sensitive life experiences, in Proceedings of the 9th Nordic Conference on Human-Computer Interaction (Gothenburg), 1–3. 10.1145/2971485.2987673

[B15] Hilvert-Bruce Z. Neill J. T. Sjöblom M. Hamari J. (2018). Social motivations of live-streaming viewer engagement on Twitch. Comput. Hum. Behav. 84, 58–67. 10.1016/j.chb.2018.02.013

[B16] Hoffner C. Buchanan M. (2005). Young adults' wishful identification with television characters: the role of perceived similarity and character attributes. Media Psychol. 7, 325–351. 10.1207/S1532785XMEP0704_2

[B17] Hu M. Zhang M. Wang Y. (2017). Why do audiences choose to keep watching on live video streaming platforms? An explanation of dual identification framework. Comput. Hum. Behav. 75, 594–606. 10.1016/j.chb.2017.06.006

[B18] Iacovides I. Mekler E. D. (2019). The role of gaming during difficult life experiences, in Proceedings of the 2019 CHI Conference on Human Factors in Computing Systems (Glasgow), 1–12. 10.1145/3290605.3300453

[B19] Johnson M. R. (2018). Inclusion and exclusion in the digital economy: disability and mental health as a live streamer on Twitch.tv. Inform. Commun. Soc. 22, 506–520. 10.1080/1369118X.2018.1476575

[B20] Johnson M. R. Woodcock J. (2017). “It's like the gold rush”: the lives and careers of professional video game streamers on Twitch.tv. Inform. Commun. Soc. 22, 336–351. 10.1080/1369118X.2017.1386229

[B21] Kosa M. Uysal A. (2020). Four pillars of healthy escapism in games emotion regulation, mood management, coping, and recovery, in Game User Experience And Player-Centered Design, ed Bostan B. (Cham: Springer), 63–76. 10.1007/978-3-030-37643-7_4

[B22] Kowert R. Domahidi E. Quandt T. (2014). The relationship between online video game involvement and gaming-related friendships among emotionally sensitive individuals. Cyberpsychol. Behav. Soc. Netw. 17, 447–453. 10.1089/cyber.2013.0656 24660878 PMC4080869

[B23] Lenhart A. Purcell K. Smith A. Zickuhr K. (2010). Social Media & *Mobile Internet Use Among Teens and Young Adults*. Washington, DC: Pew Internet & American Life Project.

[B24] Lim J. S. Choe M.-J. Zhang J. Noh G.-Y. (2020). The role of wishful identification, emotional engagement, and parasocial relationships in repeated viewing of live-streaming games: a social cognitive theory perspective. Comput. Hum. Behav. 2020:106327 10.1016/j.chb.2020.106327

[B25] Mahmoud J. S. R. Staten R. T. Hall L. A. Lennie T. A. (2012). The relationship among young adult college students' depression, anxiety, stress, demographics, life satisfaction, and coping styles. Issues Ment. Health Nurs. 33, 149–156. 10.3109/01612840.2011.632708 22364426

[B26] Massimi M. Dimond J. P. Le Dantec C. A. (2012). Finding a new normal: the role of technology in life disruptions, in Proceedings of the ACM 2012 Conference on Computer Supported Cooperative Work (Seattle, WA), 719–728. 10.1145/2145204.2145314

[B27] Nakandala S. C. Ciampaglia G. L. Su N. M. Ahn Y.-Y. (2017). Gendered conversation in a social game-streaming platform, in Eleventh International AAAI Conference on Web and Social Media (Montreal, QC).

[B28] Olson C. K. (2010). Children's motivations for video game play in the context of normal development. Rev. Gen. Psychol. 14, 180–187. 10.1037/a0018984

[B29] Pals J. L. (2006). Narrative identity processing of difficult life experiences: pathways of personality development and positive self-transformation in adulthood. J. Pers. 74, 1079–1110. 10.1111/j.1467-6494.2006.00403.x 16787429

[B30] Paulhus D. L. Vazire S. (2007). The self-report method, in Handbook of Research Methods in Personality Psychology, Vol. 1, eds Robins R. W. Chris Fraley R. Kruegerüity R. F. (New York, NY: The Guilford Press), 224–239.

[B31] Ptacek J. T. Smith R. E. Dodge K. L. (1994). Gender differences in coping with stress: when stressor and appraisals do not differ. Pers. Soc. Psychol. Bull. 20, 421–430. 10.1177/0146167294204009

[B32] Rice S. M. Purcell R. McGorry P. D. (2018). Adolescent and young adult male mental health: transforming system failures into proactive models of engagement. J. Adolesc. Health 62, S9–S17. 10.1016/j.jadohealth.2017.07.024 29455724

[B33] Rozin P. Royzman E. B. (2001). Negativity bias, negativity dominance, and contagion. Pers. Soc. Psychol. Rev. 5, 296–320. 10.1207/S15327957PSPR0504_2

[B34] Sjöblom M. Hamari J. (2017). Why do people watch others play video games? An empirical study on the motivations of Twitch users. Comput. Hum. Behav. 75, 985–996. 10.1016/j.chb.2016.10.019

[B35] Sjöblom M. Törhönen M. Hamari J. Macey J. (2019). The ingredients of Twitch streaming: affordances of game streams. Comput. Hum. Behav. 92, 20–28. 10.1016/j.chb.2018.10.012

[B36] Taylor T. (2018). Watch Me Play: Twitch and the Rise of Game Live Streaming. Princeton, NJ: Princeton University Press 10.2307/j.ctvc77jqw

[B37] Valkenburg P. M. Peter J. (2009). Social consequences of the internet for adolescents: a decade of research. Curr. Direct. Psychol. Sci. 18, 1–5. 10.1111/j.1467-8721.2009.01595.x

[B38] Wohn D. Y. Freeman G. McLaughlin C. (2018). Explaining viewers' emotional, instrumental, and financial support provision for live streamers, in Proceedings of the 2018 CHI Conference on Human Factors in Computing Systems (Montreal, QC), 1–13. 10.1145/3173574.3174048

