# Supplementary material for: Live Streams on Twitch Help Viewers Cope With Difficult Periods in Life
Source: Front Psychol. 2020 Nov 20;11:586975. doi: 10.3389/fpsyg.2020.586975 (PMC7714943; doi:10.3389/fpsyg.2020.586975)
Supplement: Supplementary file 1 [file Table_1.DOCX]

Questionnaire on Twitch Use During Difficult Times

# Informed Consent

Twitch as a coping mechanism

We would like to ask you to participate in this research into Twitch. This research is conducted under the supervision of Tilburg University and is part of a Master thesis into the support Twitch can offer someone who is coping with a difficult period in life. Before you decide to participate in this study, we will first explain how it is structured.

The research will consist of a number of questions about yourself and questions about using Twitch. However, it is important that you use Twitch and that you have experienced a difficult life period. This difficult period in life can be a stressful, confusing, troubled, or discouraging time in your life. This can for example be a divorce, depression or experiencing an illness. You can disclose as little or as much information as you want. In addition, a minimum age of 16 is required for participation.

The survey will take approximately 10 minutes to complete. Participation is voluntary and you can stop the survey at any time without any negative consequences and without providing any explanation. All your data will be processed anonymously, and your data will only be used for this research. The research data will be stored for 10 years.

This research is approved by the Research Ethics and Data Management Committee (REDC). If you have any remarks or complaints regarding this research, you may also contact the “Research Ethics and Data Management Committee” of Tilburg School of Humanities and Digital Sciences via [anon]. It is also possible to contact us directly in case you have any questions:

[researchers]

By clicking the agreement box, you confirm that you have read, and agree to all of the information above. We ask you to continue to the next page to participate in the survey and to give us permission to process your data anonymously. The consent only applies to the duration of this study.

I agree

I don’t want to participate

This is your anonymous survey ID: […]

You can write this down in case you want your data to be removed in the future. Please click the next button to go to the survey.

# Demographic Information

2.1 What is your age?

2.2 What is your gender?

Male

Female

Other

Do not wish to disclose

2.3 What is your highest level of education?

No education

Primary school

High school

Bachelor’s degree

Master’s degree

Other

2.4 In what country were you born?

# Twitch Habits

3.1 Please assess the following sentences about yourself ^1^

|  | Strongly disagree | Somewhat disagree | Neither agree nor disagree | Somewhat agree | Strongly agree |
| --- | --- | --- | --- | --- | --- |
| I define myself as a gamer |  |  |  |  |  |
| Others define me as a gamer |  |  |  |  |  |
| My friends are gamers |  |  |  |  |  |
| I watch the same games on Twitch as the games that I play |  |  |  |  |  |

3.2 How many hours do you spend watching Twitch weekly? ^1^

0–3 hours

4–7 hours

7–10 hours

+10 hours

3.3 How many days a week do you watch Twitch?

1–3 days

4–5 days

6–7 days

3.4 Which are your favorite gaming genres to watch on Twitch? (Multiple answers possible) ^1^

Action-Adventure

Adventure

MMORPG

MOBA

Real Time Strategy

Turn Based Strategy

Simulation

Racing

Survival

Western RPG

Japanese RPG

Singleplayer FPS

Multiplayer FPS

Sports

Arcade

Social Games

Puzzle games

Serious games

Battle Royale

Creative

In Real Life

Other

3.5 On average, how many streamers are present in the streams you watch? (Multiple answers possible)

0–100

100–500

500–1000

1000–5000

5000–10.000

10.000–50.000

50.000+

3.6 How often do you watch a male/female streamer?

|  | Never | Sometimes | About half the time | Most of the time | Always | I don’t know |
| --- | --- | --- | --- | --- | --- | --- |
| Male streamer |  |  |  |  |  |  |
| Female streamer |  |  |  |  |  |  |

3.7 How would you rate your personal connection to the … when watching Twitch?

|  | Very weak | Weak | Average | Strong | Very strong |
| --- | --- | --- | --- | --- | --- |
| Other viewers |  |  |  |  |  |
| Streamer |  |  |  |  |  |

3.8 How often do you use the chat function while using Twitch?

|  | Never | Sometimes | About half the time | Most of the time | Always |
| --- | --- | --- | --- | --- | --- |
| Reading |  |  |  |  |  |
| Chatting |  |  |  |  |  |

3.9 How much interaction takes place between the streamer and the viewers in the streams you watch?

There is constant interaction

There is a lot of interaction

There is some interaction

There is little interaction

There is no interaction

3.10 I watch a certain stream on Twitch…

|  | Strongly disagree | Somewhat disagree | Neither agree nor disagree | Somewhat agree | Strongly agree |
| --- | --- | --- | --- | --- | --- |
| Because of the streamer |  |  |  |  |  |
| Because of the game that is played |  |  |  |  |  |

3.11 While you are watching a play session on Twitch, what do you usually do? (e.g. just watching, commenting, talking about personal things, talking about the stream, …) ^1^

3.12 I use Twitch to… ^1^

|  | Strongly disagree | Somewhat disagree | Neither agree nor disagree | Somewhat agree | Strongly agree |
| --- | --- | --- | --- | --- | --- |
| be entertained |  |  |  |  |  |
| be informed about current game culture |  |  |  |  |  |
| avoid boredom |  |  |  |  |  |
| criticize a streamer |  |  |  |  |  |
| communicate with other viewers through the chat |  |  |  |  |  |
| find new people to play with |  |  |  |  |  |
| be part of a community |  |  |  |  |  |
| support a streamer |  |  |  |  |  |
| get in touch with a streamer |  |  |  |  |  |
| annoy other users |  |  |  |  |  |
| learn new gaming strategies and techniques |  |  |  |  |  |
| follow specific streamers |  |  |  |  |  |
| follow specific games |  |  |  |  |  |
| make friendships with new people |  |  |  |  |  |

# Difficult period in life

The following questions will entail the difficult period that you have experienced. If you do not feel comfortable continuing, you are free to stop the questionnaire at any given time. We would like to emphasize that all data is treated anonymously. All of us have times of — perhaps ongoing — personal difficulty. Please think of a stressful, confusing, troubled, or discouraging time in your life. ^2^

4.1 How would you categorize this difficult period in life? (e.g. depression, divorce/separation, physical health, …)

4.2 If you would like to elaborate, you can describe this difficult time in your life. What did you experience as stressful, confusing, troubling, or discouraging? (Optional) ^2^

4.3 Are you currently in this difficult life period?

Yes

Maybe

No

Do not wish to disclose

# Twitch during the difficult period in life

5.1 Did you watch Twitch during this difficult period in life?

Yes

No

Please bear in mind the difficult period in life while answering the following questions.

5.2 Do you think watching Twitch was helpful during this period?

Not helpful at all

Not helpful

Somewhat helpful

Helpful

Very helpful

5.3 Did you spend more or less time watching Twitch during this period in your life compared to “normal life”?

Less

A little less

Equal

A little more

More

Do not recall

5.4 What has Twitch meant for you during this period? (e.g. distraction, positivity, belonging to a community, finding a place to talk, …)

5.5 If possible, explain a specific situation in which Twitch was helpful during this difficult period in life (e.g. name of the streamer, game that was being played, your feelings, …)

5.6 On average, how many viewers were present in the streams you watched during this difficult period in your life? (Multiple answers possible)

0–100

100–500

500–1000

1000–5000

5000–10.000

10.000–50.000

50.000+

5.7 How often did you watch a … during this difficult period in life?

|  | Never | Sometimes | About half the time | Most of the time | Always | I don’t know |
| --- | --- | --- | --- | --- | --- | --- |
| Male streamer |  |  |  |  |  |  |
| Female streamer |  |  |  |  |  |  |

5.8 How would you rate your personal connection to the … during this difficult period in life?

|  | Very weak | Weak | Average | Strong | Very strong |
| --- | --- | --- | --- | --- | --- |
| Other viewers |  |  |  |  |  |
| Streamer |  |  |  |  |  |

5.9 How often did you use the chat function during the difficult period in your life while using Twitch?

|  | Never | Sometimes | About half the time | Most of the time | Always |
| --- | --- | --- | --- | --- | --- |
| Reading |  |  |  |  |  |
| Chatting |  |  |  |  |  |

5.10 How much interaction takes place between the streamer and the viewers in the streams you watch during the difficult period in life?

Constant interaction

A lot of interaction

Some interaction

Little interaction

No interaction

# End of survey

6.1 Is there anything else you would like to add? (Optional)

We would like to thank you for participation in the study. If you are willing to answer any follow-up questions regarding the research, you can leave your e-mail address in the box below. We will send you some questions via e-mail. If you have any questions regarding the survey, please contact [researchers].

^1^ Based on Gandolfi (2016)

^2^ Based on Iacovides & Mekler (2019)
